# Implementation of a fee-based service model to university-affiliated researchers at the University of Alberta

**DOI:** 10.5195/jmla.2019.497

**Published:** 2019-04-01

**Authors:** Janice Yu Chen Kung, Thane Chambers

**Affiliations:** Public Services Librarian, John W. Scott Health Sciences Library, University of Alberta–Edmonton, Canada, janice.kung@ualberta.ca; Research Impact Librarian, University of Alberta–Edmonton, Canada, thane@ualberta.ca

## Abstract

**Background:**

There is growing demand for specialized services in academic libraries, including supporting systematic reviews and measuring research impact.

**Study Purpose:**

The John W. Scott Health Sciences Library implemented a fee-based pilot project for the Faculty of Nursing for one year to test a fee-based model for specialized services, to evaluate its sustainability and scalability for the longer term, and to assess the feasibility of extending this service model to other health sciences faculties.

**Case Presentation:**

We describe the development and delivery of the fee-based service model. Through a team-based approach, we successfully provided specialized services including mediated literature searching, research support, and research impact analyses to the Faculty of Nursing.

**Discussion:**

Despite some challenges in developing and implementing the fee-based service model, our pilot project demonstrated demand for fee-based specialized services in the health sciences and suggests potential for this unique service model to continue and expand.

## BACKGROUND

Fee-based services were established in North American research libraries as early as 1955 and increased in number through the 1990s [1]. Although libraries have traditionally provided free access to information and services, the implementation of fee-based models in academic libraries has primarily been driven by demands for specialized services, the need for coordinated library services to accommodate external users, and libraries’ capacity to use these models to generate revenue [1]. However, there is also conflict in the literature between those who have a philosophical opposition to money changing hands for library services [2] and those who believe that as the landscape changes for libraries, especially around budgetary constraints and new service demands from users, it may be prudent for libraries to find revenue-generating solutions to support preexisting service models and to highlight the specialized expertise and resources available from the library [2, 3].

Many academic libraries have provided fee-based services for research support, book loans, photocopying, and document delivery for non-primary user groups of their institutions prior to the Internet age. There are also many descriptions of specialized fee-based services such as patent and commercial database searching for external communities, such as business firms, law firms, and independent researchers [1]. When access to the Internet became more readily available, demand declined for fee-based services [1], and recent searches of the literature resulted in only a handful of articles about fee-based library services since the 1960s. These included services offered to non-university clientele [4], university clientele, and a university department [5], highlighting library resources and librarians’ expertise. More relevant to health sciences librarianship, a fee-based systematic review service was implemented at George T. Harrell Health Sciences Library at Penn State College of Medicine in 2013 and was associated with several challenges related to resources, time projections, and staffing [6]. However, this service also encouraged faculty to pursue grants to fund projects and highlighted librarians’ expertise.

At the University of Alberta Libraries (UAL), a research librarian was employed in a term contract position to provide specialized library services to the Faculty of Nursing (FON) from 2008 to 2015. Although the librarian was an employee of the UAL, the FON wholly funded this embedded librarian contract position. The specialized services provided by the librarian included mediated searches, systematic review support, and research impact services. This position supplemented the large array of core library services that were already available to FON through the John W. Scott Health Sciences Library’s existing liaison program.

When the contract for this unique embedded librarian service model ended in August 2015, the FON encouraged the library to develop a fee-based service model to provide ongoing support for measuring research impact, providing mediated searches, and providing priority support for systematic review searching—essentially to replace the work the librarian did. The FON and its faculty members indicated a willingness and expectation to pay for specialized services provided by the Scott Library that were not available under the preexisting service model. As a result, in consultation with the FON, the library team developed a fee-based services model that reflected the needs expressed by FON researchers and the faculty’s associate dean of research.

## STUDY PURPOSE

A pilot was implemented to provide specialized library services for the FON from September 2015 to August 2016. This pilot project was designed to test the Scott Library’s ability to provide specialized fee-based services without interrupting core library services. Any health librarian could complete requests and provide suggestions or recommendations for difficult cases, reflecting the library’s team-based approach. As the pilot progressed, we assessed its sustainability and scalability by interviewing users of the fee-based services and library staff involved in providing the services.

## CASE PRESENTATION

In the summer of 2015, we spent significant time and effort to develop a plan for implementing fee-based services. One of our first tasks was to distinguish between specialized services that would be provided for a fee and core services that have always been, and would continue to be, offered for free to library users. Challenges to this planning process included discussions among librarians across the UAL about the ethical implications of charging for services, including the implications of two-tiered services and the equity of library services for all health sciences faculties.

Because libraries are traditionally committed to providing services for free rather than supporting those who are able to pay, there were concerns about the perception of providing specialized services to faculties with greater financial resources. Librarians working in non-health-liaison areas, in particular, pointed out that in subject areas that do not receive as much funding as science, technology, engineering, and medicine (STEM) fields, faculty members may be less able to utilize fee-based services if they were offered to the entire campus. Ultimately, we differentiated between core services and fee-based services (Table 1).

**Table 1:**
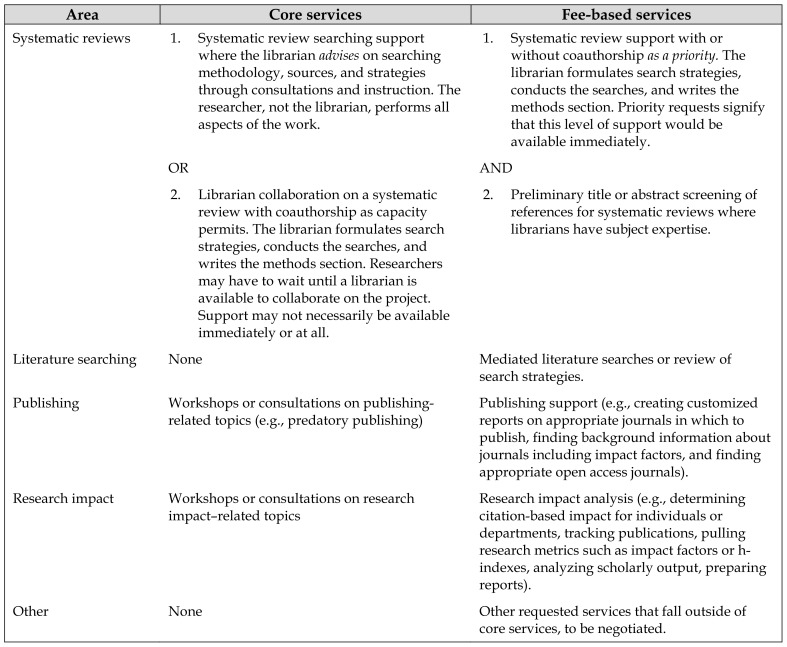
Core services versus fee-based services

Area	Core services	Fee-based services
Systematic reviews	1. Systematic review searching support where the librarian *advises* on searching methodology, sources, and strategies through consultations and instruction. The researcher, not the librarian, performs all aspects of the work.	1. Systematic review support with or without coauthorship *as a priority*. The librarian formulates search strategies, conducts the searches, and writes the methods section. Priority requests signify that this level of support would be available immediately.
	OR	AND
	2. Librarian collaboration on a systematic review with coauthorship as capacity permits. The librarian formulates search strategies, conducts the searches, and writes the methods section. Researchers may have to wait until a librarian is available to collaborate on the project. Support may not necessarily be available immediately or at all.	2. Preliminary title or abstract screening of references for systematic reviews where librarians have subject expertise.
Literature searching	None	Mediated literature searches or review of search strategies.
Publishing	Workshops or consultations on publishing-related topics (e.g., predatory publishing)	Publishing support (e.g., creating customized reports on appropriate journals in which to publish, finding background information about journals including impact factors, and finding appropriate open access journals).
Research impact	Workshops or consultations on research impact–related topics	Research impact analysis (e.g., determining citation-based impact for individuals or departments, tracking publications, pulling research metrics such as impact factors or h-indexes, analyzing scholarly output, preparing reports).
Other	None	Other requested services that fall outside of core services, to be negotiated.

The fee structure for specialized services was developed based on the previous work completed by the embedded research librarian for the FON in combination with consultations with librarians at Scott Library. We decided that the final fee structure would be CDN$100 per hour or CDN$500 per day. If the work required more than 5 hours in a day, the invoice would only be CDN$500, even if it required 7 or 8 hours of work. Researchers requested librarian support in their grants for systematic reviews in amounts ranging from CDN$500 to CDN$10,000, depending upon the amount of the grant and the complexity of the review. With grants under CDN$10,000, librarian services would be reduced to CDN$500, whereas larger grants required up to CDN$10,000. To ensure that specialized services were available to all researchers, we did not differentiate their commitment to or efforts on projects based on the size of its grant funding.

The fee structure was approved by UAL administration in July 2015. Following approval, we met with the library financial services office to develop invoicing procedures. Upon implementation of the fee-based services in the fall of 2015, we communicated extensively with FON faculty members about core (i.e., free) and fee-based (i.e., specialized) library services through email, drop-in sessions, and presentations. This service was specifically targeted to faculty members with grants. Other requests from researchers working on non-funded research projects or graduate students would continue to be served by the library’s core services.

In alignment with the UAL’s team-based approach to services, we decided that the fee-based services would be provided by a team of library staff members with varying levels and areas of expertise. As demand for fee-based services rose, the number of staff members involved increased accordingly. By the end of the pilot, five staff members supported the fee-based services model: the head librarian, a contracted librarian, two librarians on an ad hoc basis, and one paraprofessional. The head librarian oversaw administrative responsibilities, and the contracted librarian dedicated most of her time to providing the fee-based services, including issuing invoices. The two other librarians and paraprofessional filled some requests for fee-based services in addition to their normal responsibilities.

Fee-based services used by FON faculty during the pilot included eight mediated literature searches (e.g., intimate partner violence, use of emergency departments by the elderly), three research impact analysis reports, one peer review of a search strategy, and year-round services to a national research group (e.g., monitoring new scholarly publications and media outlets related to their research area, updating their blog on a biweekly basis) that fell outside of the library’s core services and were negotiated with the principal investigator, who was an FON faculty member. This work for the national research group required an average of six hours a week.

There was no set timeline for when invoices were issued to faculty for services completed. Depending on the project and terms negotiated with faculty members, some invoices were issued and paid prior to the work being completed, whereas other invoices were issued after the work was completed. Although the purpose of the pilot was not cost-driven, the library generated revenue of CDN$41,500 during the 1-year period. All revenue went to central library operations and was not retained by the health sciences library.

To determine the effectiveness of the fee-based service model, the fifteen faculty members who used this service were interviewed or emailed questions to elicit feedback. Sample questions asked of FON faculty members who used the fee-based services are:

- How useful did you find the fee-based service?
- Did the fee-based service meet your specific research needs?
- Were there research needs that the fee-based service did not meet?
- Do you have recommendations for how the fee-based service could be improved?

Overall, FON faculty who utilized the fee-based services provided positive feedback. Some comments from faculty indicated that “it was helpful to do a comprehensive search with expert librarians” and that they would use the service again. Another reported that “the process and product went really well” and the pilot “surpassed expectations.” One faculty member noted that the fee-based services offered huge value to researchers at low cost and that “researchers need to get into the habit of factoring [library-related] cost[s] into research.”

Library service providers were also interviewed. UAL librarians considered the fee-based service model a way for the library to expand its services, believing that it offered researchers new options to do their work and provided more exposure of the library and librarians’ skills to faculty members, thereby leading to greater recognition and appreciation of librarians’ expertise and research support in the university community. However, we also noted some limitations of the fee-based services model. Specifically, we received a smaller number of requests from FON faculty than anticipated, and it was sometimes difficult to clearly communicate the difference between core and fee-based services to faculty members. Upon reviewing all feedback from stakeholders, the consensus among library staff involved in the fee-based service pilot project was that the “service should continue and be expanded outside of nursing.”

## DISCUSSION

Our one-year pilot of a fee-based services model for FON indicated that this model was sustainable with a full-time contracted librarian to provide capacity for the Scott Library team to meet the demand for both specialized and core services. However, several major challenges were identified in developing and implementing the fee-based service model:

- Extensive time was required for planning, developing, and delivering the services. Setting up the administrative and invoicing infrastructure was difficult, as historically, libraries did not invoice faculty for research support services.
- Internal and external communications were problematic throughout the year. Some FON researchers remained unclear regarding the difference between core and fee-based library services, even when presented with information explaining that fee-based services were new services that supplemented the core services that they had always received.
- Competing priorities for providing core library services versus specialized services was also problematic at certain times of the academic year, especially at the start of the semester when extensive library instruction occurs.
- One of the biggest challenges was the extensive time required to complete some aspects of the contracted work. For certain projects, the librarian involved in the work was required to block off time and was unavailable to provide core services in order to meet project deadlines.

To continue meeting the demand for fee-based services, improvements were made on an ad hoc basis throughout the pilot year to simplify processes and minimize some services. One of the major administrative hurdles that required streamlining was the invoicing process. With several librarians doing fee-based work, the original process had each librarian draft an invoice for completed work for the library’s financial services. This confusing, multistep invoicing process was streamlined by designating the contract librarian as the only point of contact for all invoicing between the client and the library’s financial services. Once this change was implemented, the number of errors and inquiries between financial services and health sciences librarians was reduced.

Another issue is that research impact analyses require comprehensive retrieval of author publications. However, as some databases (e.g., Google Scholar) have poor author name disambiguation systems, we decided to retrieve author information only from Scopus, which employs a better author name disambiguation system. Streamlining some services in this manner created efficiencies and enabled scalability to potentially extend the fee-based model to other faculties.

During the pilot year, there were several unsolicited requests for specialized fee-based services from other health sciences faculties, demonstrating the potential for service expansion beyond the FON. Based on the success of the service development and delivery model, feedback from service users and providers, and potential demand from other disciplines in the health sciences, the pilot was extended from 2016–2017 and expanded to include the Faculty of Medicine and Dentistry without having to increase the number of library staff involved in fee-for-service work.

During the second year of the pilot, we revised certain parts of the fee structure to better reflect the amount of work required to meet FON demands for research impact measurement. When the second pilot year ended, the fee-based service model was implemented as a continuing service across all health sciences faculties. Our experience indicated that a business plan and a thorough evaluation strategy would help establish and monitor objectives and staffing requirements, determine the scalability of the model, and predict future growth. The evaluation conducted after the first pilot year could have been better executed if discussions occurred at the beginning of the pilot project, rather than the end, to identify the information that should be collected for assessment purposes. Marketing and communication strategies can also help inform potential users and ensure that both library staff and faculty members have a clear understanding of the services being offered.

The concept of fee-based services in academic environments is not new. Some universities offer fee-based document delivery services [7], corporate research services [4], and, more recently, systematic review support [6]. This case study extends the current knowledgebase by describing a fee-based service model that offers a broader scope of research services, including mediated literature searches, systematic review support, and research impact analyses. The flexibility of the service model allows new services to be negotiated in order to meet researcher needs.

Libraries should be responsive as new demands emerge in academia. Implementing a fee-based service model is a viable option for specialized services that have not traditionally been provided in academic libraries. Experiences at the Scott Library indicate that a clearly defined scope of fee-based versus core services and clear communication with library staff and faculty about the purpose and intended outcomes of a fee-based service model can help meet the emerging needs of faculty members.
